# History of otorhinolaryngology in Germany before 1921

**DOI:** 10.1007/s00106-021-01046-9

**Published:** 2021-04-13

**Authors:** Albert Mudry, Robert Mlynski, Burkhard Kramp

**Affiliations:** 1 School of Medicine, Department of Otolaryngology, Head & Neck Surgery, Stanford University, 801 Welch Road, Stanford, USA; 2 Klinik und Poliklinik für Hals-Nasen-Ohrenheilkunde, Kopf- und Halschirurgie “Otto Körner”, Universitätsmedizin Rostock, Doberaner Str. 137–139, 18057 Rostock, Germany

**Keywords:** Otolaryngology, Anniversaries and special events, Societies, Endoscopy, Medical specialties, Otolaryngologie, Jahrestage und spezielle Ereignisse, Gesellschaften, Endoskopie, Medizinische Fachgebiete

## Abstract

In 2021, the German Society of Otorhinolaryngology, Head and Neck Surgery is celebrating the 100th anniversary of its foundation. The aim of this article is to present the main inventions and progress made in Germany before 1921, the date the society was founded. Three chronological periods are discernible: the history of otorhinolaryngology (ORL) in Germany until the beginning of the 19th century, focusing mainly on the development of scattered knowledge; the birth of the sub-specialties otology, laryngology (pharyngo-laryngology and endoscopy), and rhinology in the 19th century, combining advances in knowledge and implementation of academic structures; and the creation of the ORL specialty at the turn of the 20th century, mainly concentrating on academic organization and expansion. This period was crucial and allowed for the foundation of the German Society of Otorhinolaryngology, Head and Neck Surgery on solid ground. Germany played an important role in the development and progress of ORL internationally in the 19th century with such great contributors as Anton von Tröltsch, Hermann Schwartze, Otto Körner, Rudolf Voltolini, and Gustav Killian to mention a few.

## Abstract (de)

Im Jahr 2021 feiert die Deutsche Gesellschaft für Hals-Nasen-Ohren-Heilkunde, Kopf- und Hals-Chirurgie den 100. Jahrestag ihrer Gründung. Ziel der vorliegenden Arbeit ist es, die wesentlichen Erfindungen und Fortschritte darzustellen, die vor 1921, dem Jahr, in welchem die Gesellschaft gegründet wurde, in Deutschland gemacht wurden. Es lassen sich 3 chronologische Phasen unterscheiden: die Geschichte der Hals-Nasen-Ohren-Heilkunde in Deutschland bis zu Beginn des 19. Jahrhunderts, wobei der Schwerpunkt v. a. auf der Fortentwicklung des verstreut vorhandenen Wissens lag; die Geburtsstunde der Subspezialisierungen Otologie, Laryngologie (Pharyngolaryngologie und Endoskopie) und Rhinologie im 19. Jahrhundert, in dem die Wissensfortschritte mit der Implementierung akademischer Strukturen kombiniert wurden; und die Entstehung des Fachgebiets der Hals-Nasen-Ohren-Heilkunde zu Beginn des 20. Jahrhunderts, in dem man sich hauptsächlich auf die akademische Organisation und Expansion konzentrierte. In dieser entscheidenden Phase wurde die Gründung der Deutschen Gesellschaft für Hals-Nasen-Ohren-Heilkunde, Kopf- und Hals-Chirurgie auf einer tragfesten Grundlage möglich. Deutschland spielte bei der Entwicklung und dem Fortschritt der Hals-Nasen-Ohren-Heilkunde international im 19. Jahrhundert eine bedeutende Rolle mit solch großartigen Mitwirkenden wie Anton von Tröltsch, Hermann Schwartze, Otto Körner, Rudolf Voltolini und Gustav Killian, um nur einige zu nennen.

In 1921, the German Society of Otorhinolaryngology, Head and Neck Surgery was founded under the name “Society of German Otorhinolaryngology Physicians” (*Gesellschaft deutscher Hals-Nasen-Ohrenärzte*). After 100 years, the time has come to return to the origins of otorhinolaryngology in Germany before the foundation of the society. It is a fitting way to highlight the necessity and the importance of this society at the time of its foundation.

Like many other specialties, otorhinolaryngology (ORL) is a medical specialty born at the end of the nineteenth century [1]. At that time, division of labor became necessary, notably for organs needing special instrumentation, thus giving birth to otology and laryngology. Progressively, otolaryngology resulted from the congruence of these two new specialties, and was rapidly associated with rhinology to become otorhinolaryngology, leading to a more complete specialty, a “harmonic triad” [2].

The early otologists were surgeons who were used to the scalpel and trephine, while the early laryngologists were physicians who combined their knowledge of the larynx with that of the chest. The link between the two, namely, rhinology, was embraced by the laryngologists. The two separate disciplines had their own practitioners, departments, and journals and only united, with some exceptions, in the first decades of the twentieth century.

The union of the specialties varied from one European country to another. Prior to the late nineteenth century, individual clinical disciplines were named after the organ they focused on. The term “otology” appears to have come into use in the mid-seventeenth century, with the Greek term *otologia*. The term “rhinology” appeared nearly a century later, first in English, and the term “laryngology” three decades later in the eighteenth century, first in French [3]. In the 1890s, the term “otolaryngology” appeared in French and the term “otorhinolaryngology” in Spanish to progressively become widely used. The association of ORL with head and neck surgery was a mid-1960s event.

In 1875, Isambert and colleagues stated: “Specialties are, in fact, one of the requirements, and we could also add, one of the real methods of progress in modern science. In medicine, as is the same everywhere, the division of labor has become a necessity. The time of acknowledgeable scholars is gone … Those organs that are not directly accessible to our senses are becoming one by one more accessible thanks to exploratory instruments … In conjunction with the diseases of the larynx, we are always obliged to describe those of the pharynx, the nasal fossa and of the mouth … For these different diseases, there is a common ground” [4]. Everything was a question of mucosa!

## Background

Otorhinolaryngology went through three chronological phases:

- *creation* in the second part of the nineteenth century;
- *consolidation* in the first part of the twentieth century, which concerns the period of study; and
- *expansion* since the second part of the twentieth century [5, 6].

The first phase of the history of ORL, creation, was marked by the foundation of the first ORL hospital departments and university lectures and chairs, the organization of the first specific national and international congresses, and the publication of the first ORL journals and books. According to Isambert et al., “The close anatomical and pathological relations existing between the ear, the nose and the throat often render it necessary that diseases of those organs be treated by the same hand” [4]. The second phase, consolidation, was particularly marked by the recognition of ORL as a medical specialty and its obligatory teaching during medical studies. Special training was organized to obtain the title of ORL specialist.

The aim of this article is to present the main German inventions and advances, but also the academic organizations that preceded and led to the creation of the German Society of Otorhinolaryngology, Head and Neck Surgery in 1921. The limitations of this study are associated with manuscript length restrictions, and perhaps with the impossibility of studying in detail all the places in Germany where ORL was practiced during this period. Another limitation is that the article will not present a complete and chronological history of ORL, but only particular facts.

The text is divided into three main sections: history of ORL in Germany until the beginning of the nineteenth century, focusing mainly on the development of knowledge; the birth of the subspecialties otology, laryngology (pharyngo-laryngology and endoscopy), and rhinology in the nineteenth century, combining the advancement of knowledge and implementation of academic structures; and the creation of the specialty ORL at the turn of the twentieth century, mainly concentrated on academic organization and expansion.

## Methodology

### Literature search

The first stage of research was to look for similar publications. Since the mid-twentieth century, the history of otorhinolaryngology, head and neck surgery (OHNS) became a subject of interest with two main levels of research: a universal overview of ORL history and its subspecialties [7, 8], and a more specific ORL national view. Two partial ORL histories were published in Germany: a 1989 publication dealing with the history of rhino-laryngology [9], and a 2003 publication compiling more than 30 historical articles related to different and particular aspects of the history of ORL [10]. There are hardly any specific details on the history of ORL in Germany until 1921 in these two textbooks. Nevertheless, they contain many relevant but dispersed details about German protagonists. Only one valuable chapter about a part of this history was found in *Die Entwicklung der medizinischen Spezialfächer an den Universitäten des deutschen Sprachgebietes* (*The Development of Medical Specialties in the Universities of German-Speaking Countries*) published in 1970 [11]. Concerning otology in Germany, specifically, a paper published in 1902 [12] and a chapter published in 1913 [13] were found. Two other German publications are of general interest, but of very limited use because they collect almost only twentieth century documentation about the academic ORL chairs [14] and ORL clinics [15] in Germany. Other more focused books are available, dealing with the history of ORL notably in Berlin [16], Munich [17], Würzburg [18], Düsseldorf [19], Berlin-Wedding [20], and Rostock [21]. A few specific chapters are also found in books dealing with the history of some German universities.

To complete these sources, at least six biographies of German physicians were found: those of Hermann von Helmholtz [22], Gustav Killian [23, 24], Friedrich Hofmann [25], Anton von Tröltsch [26], Otto Körner [27], who also published his autobiography [28], and Johannes Kessel [29]. Confronted with this paucity of publications, a second level of research was thus necessary to find additional related papers, again with not much success. Very few articles were usable, notably one dealing with the origin of the first German ORL clinics [30]. This situation explains why it was important to study this period, but also the difficulty in collecting the best available material. It can also explain why some facts are certainly missing.

## History of ORL until the beginning of the 19th century

During the first steps of development of ORL, knowledge was scattered throughout many different countries without a truly progressive line of advancement in one specific country, with some exceptions as exemplified by the main advances made in ORL anatomy in the sixteenth century in Italy. Thus, to better understand the development of ORL knowledge in Germany until the beginning of the nineteenth century, it is necessary to consider it in an overview of the general ORL history during the same period. It is outside the scope of this research to go into detail, and therefore we highlight some of the main points only [31].

The history of ORL is as old as medicine itself

The history of ORL is certainly as old as medicine itself. The first medical writings found in ancient civilizations demonstrated that the symptoms were the diseases, such as painful tongue, face ecchymosis, ear that heard badly, ear that gave water of decomposition, tumefaction of the neck, swelling in the throat, fetid nose, or exudate in the nose. The enumeration of symptoms was a fundamental step in the comprehension of the disease. Physical examination was limited to external observation and palpation, except inside the mouth. Treatments were purely empirical, based on remedies of vegetal, mineral, and animal origin, for example: oil, fat, honey, sea salt, cumin, beer foam, date wine, boiled hedgehog’s thorns, rat head, fly specks, human bone, red ground ochre, mercury, copper, arsenic, and malachite. Some surgical methods, mainly in relation to trauma, were used, such as sutures, digital reposition of the nose, as well as reconstruction of the lobes of the ear and nose with flaps. Extraction of foreign bodies from the ear, excision of the uvula, incision of throat abscesses, and nose tamponade were also mentioned.

### The Ancient Greek and Roman world

The Ancient Greek world introduced the concept that diseases were not supernatural but had a natural origin, and based on the Hippocratic theory of the four humors, each one could be insufficient or excessive. Thus, it introduced new therapies based on purgation (emetics, clysters, bloodletting, and cupping), cauterization, fumigation, modification of the ambient environment, and diet. Another important concept that influenced ORL until modern times was the idea that effusions of the ear and the nose were excretions of the brain.

The Roman world improved on humoral theory and added the concept of “disease of the parts”—i.e., organic origin of disease, as an example from the ear or the larynx. Anatomy was very superficial and saw its first descriptions, mainly based on animal studies. Many new words were introduced to name different parts, notably for the auricle, the cartilage of the nose and larynx, and the muscles of the larynx. The external auditory canal ended with a “dry thin-spun web,” the hidden part of the ear was simply named “labyrinth,” and the windpipe the “trachea-arteria.” Certain surgical techniques were clearly exposed, such as: the extraction of foreign bodies in the ear with a hook, an ear-spoon, or “auricular clyster”; the ablation of nasal polyps with a special knife or a sponge attached to a string passed into the nose, to forcibly draw the polyp from its attachment; the ablation of tonsils with a finger, or with a hook and a scalpel; and the sectioning of the uvula. From the first century BC, the opening of the trachea was clearly discussed under different appellations, such as “laryngotomy,” “cutting the larynx,” “incision of the arteria,” or “pharyngotomy.” At the same time, the first pharmacopoeia was published, which listed more than 1000 remedies, mainly plants, but also minerals and products of animal origin.

### The Middle Ages

The Middle Ages did not bring much innovation, except for some surgical instruments and the idea of a kind of “cold which arises during spring when roses deploy their perfume.” The first known bivalve ear and the first nose speculum for extracting a foreign body from the external auditory canal were described in 1368: “You may be able to expose him to the sunlight by tugging the ear to dilate it with a speculum” [32]. Bedside examination, essentially represented by anamnesis and superficial physical examination, remained definitively fundamental in the initial approach to OHNS diseases. It was refined during subsequent developments in ORL.

### The Renaissance and early Modern Age

The Renaissance and early Modern Age (sixteenth to eighteenth centuries) opened new fields in medicine, mainly in anatomy and pathology. Human dissection became possible, thus leading to the progressive description of all the macroscopic parts of the body with the introduction of numerous new terms such as “tympanic membrane,” “cochlea,” “maxillary antrum,” or the proper use of other terms such as “cricoid cartilage.” In particular, the nasal turbinates, the four sinuses of the face with their orifices, the three ear ossicles, the tympanic cavity, the vestibule, the semicircular canals, the cochlea, the detailed anatomy of the larynx, and the cranial nerves were described. The salivary glands were elegantly depicted with their respective excretory canals. Saliva was demonstrated to originate not from the lymph, as suspected before, but from these glands.

Johann Friedrich Cassebohm (1699–1743) from Halle/S. published an anatomical work on the organ of hearing, notably its embryonic part, which became the reference until the mid-nineteenth century. The accompanying drawings were the best for their time [33]. A second step was made with the introduction of the simple microscope, leading to the description of most of the details of the inner ear, and the confirmation that it is filled with fluid and not air as supposed since antiquity. The anatomist Philipp Friedrich Theodor Meckel (1756–1803) in Halle/S. added new details to the description of the aqueducts of the inner ear [34].

Human dissection became possible during the 16th–18th centuries

By the end of the seventeenth century, knowledge of the gross anatomy of the larynx was complete. There remained the addition of details of the nerve supply [35] and structure of the epithelial lining. In 1797, the anatomist Carl Samuel Andersch (1732–1777) from Göttingen posthumously published an elegant dissection of the laryngeal nerves. He displayed each of the branches of the recurrent laryngeal nerves and attributed no muscular branches to the superior laryngeal nerve, with the exception of one to the cricothyroid muscle. He traced filaments of the nerve to the mucous membrane. His name is eponymously associated with the petrosal ganglion of the glossopharyngeal nerve [36].

Physical examination expanded with the detailed study of the external auditory canal and the nostrils rendered possible with the bivalve speculum, notably improved by the German-born surgeon Guilhelmus Fabry von Hilden (1560–1634), commonly known as Hildanus. He is considered one of the first surgeons to recognize the importance of anatomy in the practice of surgery and of medicine in general, anatomy being “at once the key and the rudder of medicine.” He demonstrated this in publishing an anatomical treatise in 1624 [37]. He had a great genius for observation and his collection of 500 surgical observations published between 1606 and 1627 was the best work of its kind in the seventeenth century [38]. He notably described an ear speculum [39] and a snare for uvulectomy.

Reconstructive surgery was advanced with the improvement of flaps, particularly for the nose and the ears, and the use of prosthesis for the same parts. Tracheostomy (a term introduced at the beginning of the seventeenth century), even if not very popular, involved different techniques being performed with a vertical, horizontal, or punctiform opening, associated with the placement of a canula. The advent of pathology and the understanding of the local lesions related to the disease made it clear that defluxion of the nose and the ears came from these respective parts and not from the brain as thought since antiquity. Konrad Victor Schneider (1614–1680) from Bitterfeld played a key role, when he wrote that, “catarrhal matter is not an excrement from the brain, but a bloody mass” [40] and originated from the inflamed organ. He studied the origin of nasal discharge to definitively demonstrate that it originates from the anteroposterior pituitary membranes, i.e., the nasal mucosa. The Schneiderian membrane, also called the “Schneiderian epithelium,” the lining of the paranasal sinuses and nasal cavity, is named after him. He showed that, in fact, no fluid from the viscosity of mucus was present in the cranial cavity. Concerning ear catarrh, he also demonstrated its origin in the ear, although this was not accepted in his time, but only nearly a century later. He mentioned for the first time the adenoid: “It is of a whitish color, the adjoining membranes being bloody or dusky. It is fuller than they and like fat. It is always moist and exudes a glutinous substance” [40, 41].

Hildanus was one of the first surgeons to recognize the importance of anatomy

Günther Christoph Schelhammer (1649–1712) from Jena was the first to decisively and unequivocally take the stand that “genuine air” had no physiological significance. He argued that it could not be the “instrument of hearing” because sound was conducted by air and the medium itself could not be the sense organ, just as the lens of the eye, or vitreum, is not itself the organ of visual perception. Although he opposed the idea of “genuine air” and considered the auditory nerve as indispensable to hearing, he obscured his theory with the then popular concept of “animal spirit,” which he believed necessary for the process of hearing: “The nerve by itself has nothing to do with the perception of sound, and it could certainly be absent if this so-named animal spirit was not required for the knowledge of species [i.e. sensations] of hearing” [42]. In another passage, Günther Christoph Schelhammer wrote that: “If for example a knife or a two-pronged fork, which we use to eat, are strongly struck against the wood of a table, so that the iron shakes, and we approach the other end to the teeth, we will perceive a sound in a very elegant and very clear manner. But if we introduce it completely into the mouth, we perceive nothing.” It appears that Günther Christoph Schelhammer was probably the first to have recognized the excellent vibratory properties of forks and employed them for experimental purposes before the invention of the tuning fork in 1711.

At the same time, the surgeon Johannes Scultetus (1595–1645) from Ulm published a surgical book that had many editions and translations into different languages. He notably described the dangers associated with using an ear syringe. Antique surgeries were slightly improved and new ones were introduced: the opening of the maxillary sinus in the case of infection through three different routes—the canine fossa, the tooth, and the nasal wall of the maxillary sinus; the superficial opening of the mastoid area in the case of abscess as soon as fluctuation was felt with the trephine, or gouge and mallet, rugine, or a perforator; the catheterization of the Eustachian tube first through the mouth then through the nose with a “silver tube” in the case of obstruction of “external and internal auditory passages”; and the perforation of the tympanic membrane “with a sharp, long, but small lancet,” in the case of deafness. The surgeon Justus Arnemann (1763–1806) from Göttingen published the first textbook dealing exclusively with the perforation of the mastoid process in 1792 [43]. Pain, bleeding, and infection were the limiting factors of surgical interventions.

The major, sometimes endemic and not infrequently fatal, throat condition was suffocative angina and ulcerations, sometimes with false membrane, later grouped under the name “diphtheria” [44]. The first to discuss deviations of the nasal septum appears to have been the anatomist and surgeon Samuel Theodor Quelmaltz (1696–1758) from Leipzig, who published an essay in Latin in 1750 entitled “On the Nose and Its Septal Bendings” [45]. He considered the causes of obstruction to be pressure on the nose in a difficult labor, falls in infancy, the continual thrust of the finger into the nose in childhood, and inflammatory conditions. He did not discuss treatment of the condition, which was not considered until the following century.

Pain, bleeding, and infection were the limiting factors of surgical interventions

**Fig. 1:**
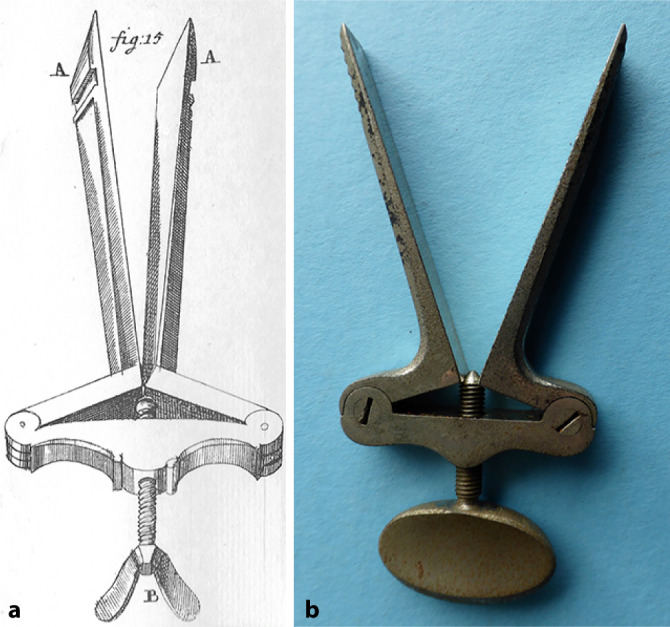
Heister mouth gap. **a** Heister’s textbook, tab. XIII, p. 532 bis [46]; **b** personal collection, Dr. Lübbers. (Reprinted with permission © W. Lübbers, Hannover, all rights reserved)

The surgeon Lorenz Heister (1683–1758) in Helmstedt wrote a general review of surgery first in 1719 [46], which also had many editions and translations. He described numerous instruments such as a mouth gap (Fig. 1). He developed a surgical technique to correct “nose holes glued against nature.” He also proposed using a lead or silver cannula placed in the nose to maintain it in case of fracture. Ludolf Heinrich Runge (1727–1756) from Bremen wrote a dissertation on diseases of the frontal sinus, maxillary sinus, and to some extent the mandible. He described an almost perfect systematics of the diseases of the paranasal sinuses, and proceeded from Konrad Schneider’s knowledge that the nose and the paranasal sinuses are lined by the same mucous membrane to conclude that any inflammation of the nasal mucosa can spread to the sinuses and lead to an accumulation of pus there. Treatment should then be drainage of the affected cavity [47].

Deaf–mute children were no longer considered as “burdensome pariahs,” and their systematic education was of public concern and established with two main controversial approaches, i.e., oralism and manualism. The systematic and methodical education of deaf–mute individuals began in Germany with Samuel Heinicke (1729–1790; [48]). In 1778 he founded the first institute for instructing deaf–mute people in Leipzig and directed it until his death. Heinicke’s methods were strictly oral. First, the pupil had to learn to take in with their eyes the meaning of what a person said, then when they understood the thought, they could be taught the various forms and symbols in which speech was expressed. Surprisingly, Heinicke never described his method in detail in his publications, but indulged in general considerations that did not give sufficient clues to his methodology. Nosologies were implemented in an attempt to classify the different known diseases, thus introducing new terms, such as “otitis” and “epistaxis.”

Otology began to be a separate topic [49] with the publication of its own books, the first in German by Christian Friedrich Ludwig Wildberg (1765–1850) from Neustrelitz (at the time Neuenstrelitz), in 1795 [50]. It was one of the first comprehensive textbooks on diseases of the ear in German. The results and discoveries in previous specialized publications, and the works of foreign authors that were difficult to obtain, were put together to form a well-organized whole. The book was divided into three parts: anatomy, physiology, and pathology.

At the turn of the nineteenth century, anatomo-pathology was implemented and completed the progress made in anatomy.

## The development of subspecialties

### Laying the foundation

#### Examination techniques and instrumentation

The first part of the nineteenth century was marked by clinico-experimentation, which progressively completed bedside examination in the understanding of ORL diseases. It made it possible to make more precise diagnoses, thus rendering specific therapy more efficient. It opened up a new approach in medical practice with the correlation of the bedside clinical symptoms and the lesions described during autopsy. Anamnesis became more orientated and more precise. A second, later step was added with the possibility of investigating the quality of the different liquids and secretions of the body in the laboratory, and also the microscopic structures of the tissues (biopsies).

New examination techniques with novel definitive instruments were introduced to directly find these lesions. The funnel-shaped speculum was finally accepted, the laryngeal mirror and the bivalve nasal speculum found their definitive shape rendering physical examination more objective. These three instruments precluded the development of the three subspecialties of otology, laryngology, and finally rhinology. The first ORL journal—after specialized otology journals were founded more than a decade earlier—was published in 1875 in French (*Annales des maladies de l’oreille et du larynx*) with the subtitle “otoscopy, laryngoscopy, rhinoscopy” [51].

#### Cell theory

Before going into more detail about these emerging subspecialties, some other overall aspects must be presented. The development of cell theory, and thereby the demonstration that most diseases were linked to cellular troubles, and that the ear, nose, and larynx have a similar lining in most of their parts (i.e., respiratory mucosa), led to the concept of a common insight in the development of these diseases. A clear relationship was demonstrated, directing physicians to join together in the care of the diseases of these organs and to create the ORL specialty. At the same time, physiological experiments began to be conducted to understand the functions of the ORL organs.

#### Advances in surgery

Up until the first part of the nineteenth century, surgery did not really progress and remained very limited. Some already described operations were demonstrated as being dangerous such as mastoidectomy. Other interventions became very popular. Clinico-experimentation reinforced anatomo-pathology for optimal-quality diagnostics.

In the first part of the nineteenth century, a new era of surgery commenced, which developed further in the second part of the century. The invention of anesthesia with ether and chloroform in the 1840s and the introduction of asepsis (sterilization), antisepsis (disinfection), and arterial clamps in the 1860s ushered in a completely new surgical era in the second part of the nineteenth century with the possibility to operate for more than only a few minutes and with fewer surgical complications. New operating rooms were created. In parallel, medication for pain relief was developed (morphine, salicin), thus, rendering postoperative care more successful and affordable for the patients. With all these surgical possibilities, ORL rapidly became a surgical specialty. New techniques were progressively introduced allowing for more precise surgery.

The discovery of bacteria as agents responsible for the development of infection was another important event. Concomitantly, local anesthesia with cocaine was introduced in 1884 [52]. It opened a new field of “minor” operations notably in the larynx and the nose. In the second part of the nineteenth century, diphtheria remained a fatal condition. The idea to pass an oral tube into the trachea to relieve airway obstruction [53, 54] represented another solution for treating this condition. This gave birth to modern intubation for general anesthesia. Tuberculosis, notably of the larynx, was another matter of concern. Specialized catalogues of instruments completed the first textbooks dealing with ORL.

#### Role of radiology

At the turn of the twentieth century, the discovery of X‑rays modified the practice of ORL. Radiology made it possible to look directly inside the ORL cavities of the face leading to the development of numerous different approaches to specifically analyze the ear, the sinus, and the larynx. The development and evolution of cancer are better understood in the ORL region, including the evaluation of primary local metastases as a part of diagnosis and treatment. Radiology also enabled the detection of swallowed or aspirated foreign bodies in the airways and esophagus. Radiotherapy (Röntgen therapy) was introduced, such as radium therapy. Radium therapy was promptly stopped because of its many side effects. All these developments found a specific place in the progress of the ORL subspecialties.

### Otology

**Fig. 2:**
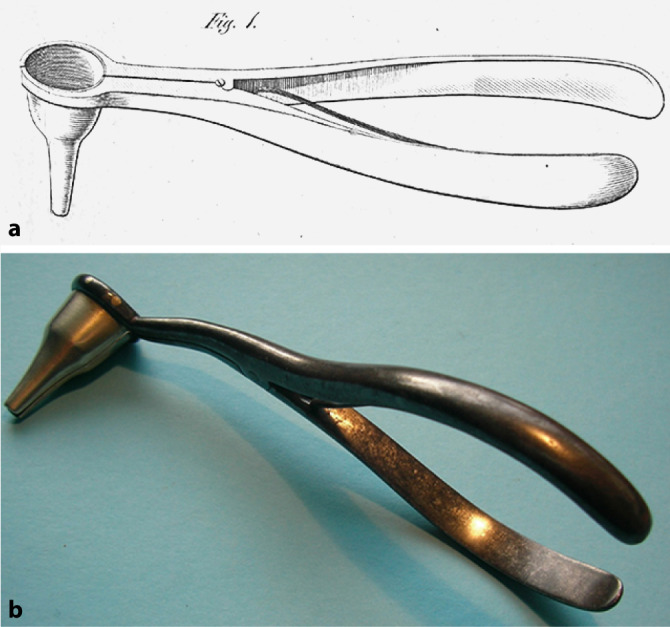
Kramer’s ear speculum. **a** from Kramer’s textbook (1836, [56]); **b** personal collection, Dr. Lübbers. (Reprinted with permission © W. Lübbers, Hannover, all rights reserved)

Often combined with ophthalmology, otology became a specialty in the first decades of the nineteenth century, notably with the opening of the first ear polyclinics and the growing number of published books dealing exclusively with the ear. First in the United Kingdom and in France, this movement took place in Germany from the 1830s [55]. Two German protagonists played an important role: Wilhelm Kramer (1801–1875) from Berlin and Carl Gustav Lincke (1804–1849) from Leipzig. For more than 40 years Kramer worked on otology and published many textbooks. His main textbook, *Die Erkenntniss und Heilung der Ohrenkrankheiten* (*The Nature and Treatment of Diseases of the Ear*; [56]), translated into French and English, saw three expanded editions. His publications occupy a special place in the otology literature of the nineteenth century. His main contribution was that the treatment of ear diseases should not depend on symptomatology, but on clinical examination of the ear. This led to a new era in otology, and prompted the development of otoscopy. However, he was a fervent adversary of pathological anatomy, and engaged with his colleagues in a very long saga of conflict. “What good work Dr. Kramer actually did for otology in his younger days has been overshadowed by his subsequent writings […] He still persists in rejecting the modern method of investigation, as well as the results of examinations of ears removed from persons who have been deaf” [57]. His name is also eponymously associated with an ear speculum (Fig. 2).

**Fig. 3:**
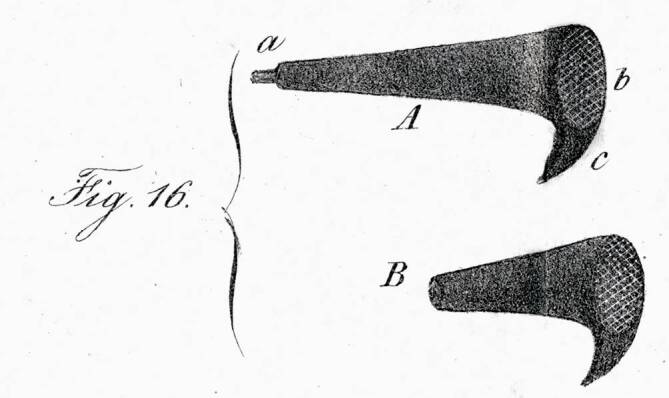
Lincke’s tympanostomy tube. (From Lincke’s textbook, 1845, [58])

Lincke published a comprehensive and well-illustrated work on otology entitled a *Handbuch der theoretischen und praktischen Ohrenheilkunde *(*Handbook of Theoretical and Practical Otology*) in three volumes between 1837 and 1845 [58]. Only the first two were written by Lincke. Illness forced him to transfer the writing of the third volume to the German otologist Philip Heinrich Wolff (1813–1886) from Berlin. He completed his otology publication with the five-volume *Sammlung auserlesener Abhandlungen und Beobachtungen aus dem Gebiete der Ohrenheilkunde* (*Compilation of Chosen Essays and Observations from the Area of the Diseases of the Ear*), in which he assembled many important otology works published in the second part of the eighteenth and essentially in the first part of the nineteenth century [59]. He translated texts not originally written into German language. Gustav Lincke’s approach contrasted sharply with that of Wilhelm Kramer, who was very critical of Gustav Lincke’s views [60]. In his comprehensive work, he carefully collected the achievements of previous otologists, attempting to save them from falling into oblivion. However, he also recognized new achievements. This was a period when historical medical studies were considered a waste of time and when Kramer declared with authority that there was nothing to be gained from studying the pathological anatomy of the ear. Gustav Lincke’s tireless enthusiasm for studying old literature therefore deserves special recognition. His handbook will always remain a valuable source for the historian. It “comprises all that was known upon the subject of aural surgery at the time in which it was written” [61]. In 1845, he developed a specific device to avoid rapid closure of the artificial tympanic perforation. Gustav Lincke explained that “the most suitable solution here is to insert a small rubber tube, with one end being so slim, that it could be inserted into the opening of the tympanic membrane. The tube is shaped to become gradually wider, and its other end is the same width as the opening of the external auditory canal” (Fig. 3; [58]) The tube was as long as the external auditory canal and its external part had a curved border to remain fastened to the tragus. In the same year, Martell Frank (1810–1886) of Munich presented another such device [62]. They were the first tympanostomy tubes that became popular only in the second part of the twentieth century [63].

To better understand the place occupied by German ear physicians in the development of otology as a specialty, it is better to follow a clinical chronology. The anatomy of the ear was improved with the use of the compound microscope, and physiology of the ear by the possibility to experiment. In 1778, the German anatomist Samuel Thomas Soemmerring (1755–1830) proposed separating the cranial nerves into 12 pairs, and to differentiate the facial and auditory nerves by giving them two different numbers, seven for the facial and eight for the auditory nerve [64]. As descriptions of the anatomy of the senses and the reproductive organs were lacking in his classic anatomical treatise, Soemmerring supplied some of them with the publication of his *Abbildungen des menschlichen Hoerorganes* (*Illustrations of the Human Auditory Organ*) in 1806 [65], which was an international reference for many years. Friedrich Christian Rosenthal (1780–1829) from Greifswald described, in 1823, the canal in the cochlea, which bears his name [66].

Known for his description of the otic ganglion, the German anatomist Friedrich Arnold (1803–1890) first mentioned in 1829 a “not described nerve” expanding “in the skin of the internal part of the external auditory canal” [67], of which he gave more details 2 years later: “This nerve going to the external ear, which could be termed an ear branch of the lung-stomach nerve (ramus auricularis nervi vagi [auricular branch of pneumogastric nerve]), springs both from the nodule of the voice nerve and from the petrous nodule, then enters the Fallopian canal and goes to the external ear” [68]. Concomitantly, he described a provoked reflex: “I think I should still say only that certain phenomena such as coughing and even vomiting can be adequately explained by touching or stimulating the external auditory canal through the ear branch of the lung-stomach nerve [pneumogastric nerve].” This was later named “Arnold’s ear cough reflex” [69] or “Arnold’s nerve cough.”

Emil Huschke (1797–1858) from Jena completed the anatomical works of Soemmerring. He published various works on embryology and paid special attention to the development of the sense organs, particularly the ear and the eye, discovering that both organs originated in a furrow-like fold of the skin. In 1835, he described the incisor-like folds in the cochlear duct of birds, and in particular the auditory teeth [70], later called “Huschke’s teeth” [71]. Huschke’s name was also associated with a second structure of the ear, the “tympanic foramen” [72]. This was a passage formed by the joining of the two prominences of the tympanic ring near the inner extremity of the tympanic portion of the temporal bone. It normally became ossified and disappeared during early childhood [73].

**Fig. 4:**
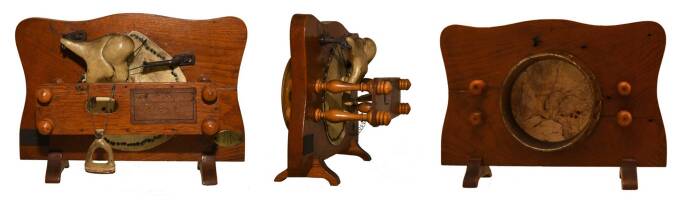
Helmholtz’s ossicular chain model. (Private collection. Reprinted with permission © A. Mudry, all rights reserved)

After the description of the hair cells and the organ of Corti in 1851 [74], Rudolf Albert von Kölliker (1817–1905) of Würzburg conducted numerous anatomical studies of the inner ear [75]. Otto Friedrich Karl Deiters (1834–1863) of Bonn, known for the most comprehensive description of a nerve cell at the time, depicted the supporting cells in the cochlea [76], later named “Deiters’ cells” [71]. It guided the reworking of the theories of hearing, notably by Hermann von Helmholtz (1821–1894) from Berlin and his concept that different regions of the basilar membrane act as resonators for tones of different frequency. His main publications were *Die Lehre von den Tonempfindungen *(*On the Sensation of Tone*; [77]), which had five editions and was translated into several languages, and *Die Mechanik der Gehörknöchelchen und des Trommelfells* (*The Mechanics of the Ossicles and Tympanic Membrane*; [78]), which led him to develop various simulation models (Fig. 4). He was certainly one of the most remarkable nineteenth-century figures in German physiology. He also moved into the sphere of electrodynamics. He was assisted by Heinrich Rudolf Hertz (1857–1894), whose discovery of “Hertzian waves” made modern wireless transmission possible. In the late 1960s, and in recognition of Hertz, his name was given to the unit of frequency—one cycle per second—and is abbreviated Hz.

Another important discovery was made regarding the physiology of the ear: that the balance organ was in the inner ear, more precisely in the semicircular canals, after the demonstration, in 1824, that in cutting the semicircular canals of birds, balance problems were described [79]. After the recognition that vertigo can stem from a pathology of the ear [80], Friedrich Leopold Goltz (1834–1902) in Halle/S. and Strasbourg went a step further in definitively demonstrating, in 1870 [81], that the balance system is in the semicircular canals. Finally, it was shown that nystagmus was a labyrinthine reflex [82, 83]. Friedrich Goltz’s assistant, Ernst Julius Richard Ewald (1855–1921; [84]), described the two Ewald’s laws [85], dealing with the movements of endolymph in the semicircular canals. These were obtained from research on pigeons, using a “pneumatic hammer.” Julius Ewald’s first law stated that the direction of endolymph flow is in the direction of the slow phase of nystagmus. His second law maintains that ampullopetal endolymph flow in the horizontal semicircular canal causes greater nystagmus response than ampullofugal endolymph flow in the horizontal canal and the reverse was the case for the vertical canals.

In 1860, Gustav Theodor Fechner (1801–1887) in Leipzig [86], introduced the term “psychophysics” and described, with Ernst Heinrich Weber (1795–1878) also in Leipzig, the Weber–Fechner law related to the nonlinear relationship between the physical intensity of a stimulus (such as sound) and the perceived sensation.

Laboratory work facilitated the recognition and the description of many diseases such as cholesteatoma [87] and otosclerosis [88]. The term “cholesteatoma” was coined in 1838 [89] by Johannes Müller (1801–1858) from Berlin because he was aware of the presence of cholesterol and fat in what he believed to be a fatty tumor. Three main theories were then discussed in Germany and abroad to explain the origin of this entity:

- metaplasia of mesenchymal cells by Rudolf Virchow (1821–1902) in Berlin [90],
- heterotopia, and
- epithelial migration of the external auditory canal epidermis into the tympanic cavity supported by Friedrich Bezold (1842–1908) of Munich [91].

It then took 40 years of discussion about these three theories to finally confirm that the migration theory was the most probable one. In 1840, Samuel Moritz Pappenheim (1811–1882) from Breslau discovered inflammatory changes in the mucous membrane of the tympanic cavity in the case of suppurative otitis resulting from typhus and pneumonia [92]. This was the first step in explaining the origin of otitis media in a modification of the mucous layer of the tympanic cavity.

The term “cholesteatoma” was created in 1838

**Fig. 5:**
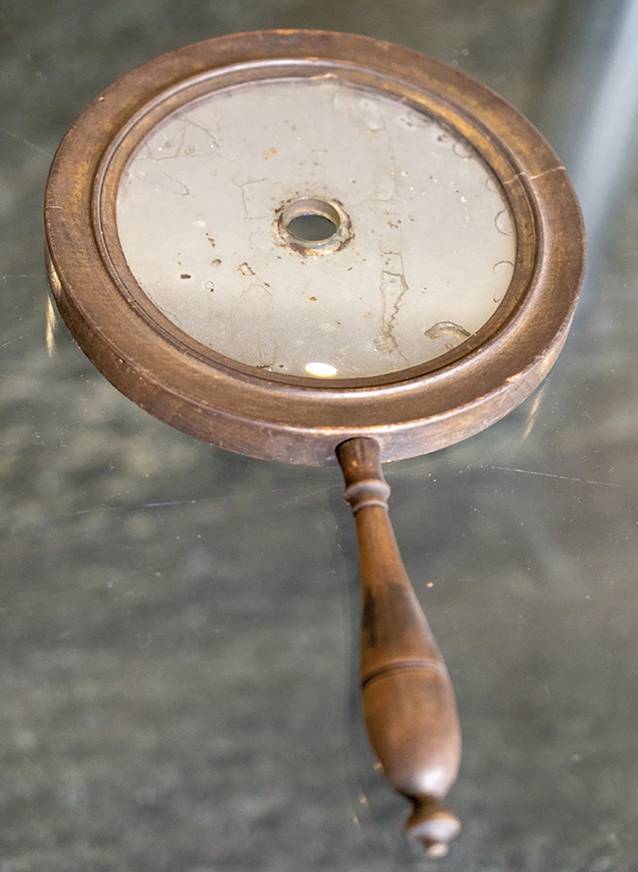
Hofmann mirror. (Würzburg ORL Clinic Collection)

**Fig. 6:**
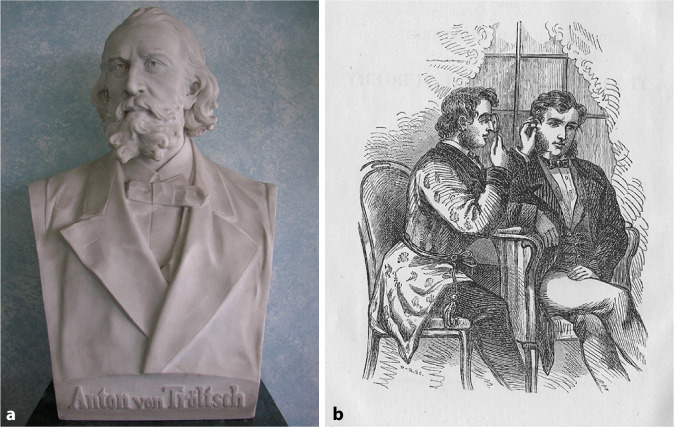
Anton von Tröltsch and his demonstration of the technique of how to use the adaptation of Hofmann’s mirror. **a** Würzburg ORL Collection; **b** from Tröltsch’s textbook, 1864. (From [279])

Clinical examination achieved its definitive landmarks when, in 1841, the Westphalian country physician (“*Landarzt*”) Friedrich Hofmann (1806–1886) from Burgsteinfurt put forward the idea of using a hand-held concave mirror for reflecting and directing sunlight or artificial light onto the ear speculum [93]. A hole was perforated in the center of the mirror to allow direct visualization by the examiner (Fig. 5). Hofmann also described a funnel-shaped speculum with three mobile branches. Anton Friedrich Freiherr von Tröltsch (1829–1890) [94] from Würzburg made this method popular by claiming its paternity (Fig. 6; [95]).^1^ He was one of the three founders (together with Adam Politzer [1835–1920] of Vienna and Hermann Schwartze [1837–1910] of Halle/S.) of the *Archiv für Ohrenheilkunde* (*Archives of Otology*), the first journal dealing with otology [96]. In 1873, he was considered as an “outstanding German otologist” [97]. He played an important role in the development of otology not only in Germany, but also abroad. His main textbook, *Die Krankheiten des Ohres *(*Treatise on the Diseases of the Ear*; [98]), had seven editions and an international audience with its translation into French, Italian, and English. He also published the first complete text on pediatric otology [99].

Emil Siegle (1833–1900) from Stuttgart invented the pneumatic otoscope [100], an otoscope that allowed the pressure in the outer auditory canal to be modified so that the mobility of the tympanic membrane could be assessed: “When examining patients with ear problems, it is of interest for the physician to obtain information regarding the mobility of the tympanic membrane […] The instrument used […] is an approximately one-inch-long by half-inch wide metal cylinder to which an ear speculum is attached at one end by means of a screw mount. The other end is closed airtight, with a piece of glass. On a part of the cylinder a perforated small attachment is located over which an approximately one-foot-long rubber tube is placed […] the free end of the rubber tubing into his mouth […] Because of the elastic cover which is placed over the ear speculum the space is airtight and communicates only with the physician’s mouth. By a gentle sucking at the rubber hose, it is possible to thin the air in the external ear canal. The ear drum suddenly moves outwards, the ossicles swing around their axes and the light reflex changes and turns broader. Immediately after releasing the suction, everything will return to its old position. All this can be observed through the occluding glass window.”

Ernst Florens Friedrich Chladni (1756–1827) of Wittenberg, often referred to as “the father of experimental acoustics,” was the first scientist to systematically investigate tuning forks, using his famous powder method to reveal the patterns of vibration on vibrating objects [107]^2^. Ernst Heinrich Weber discovered the phenomenon of the lateralization of sound by the cranium in 1834 [103]; he observed that sound from a tuning fork placed in the middle of the head, and transmitted through the bones, was perceived by both ears. He described his test as follows: “If we firmly block one ear with a hand and voice a sound, we perceive very clearly that it is heard much better and much louder by the blocked ear than by the unblocked one […] If we touch our teeth with the point of a tuning fork (oscillating musical fork) producing a not too high-pitched sound, and we block our mouth as much as possible with the lips and tongue, and at the same time close our ears either by pressing our hands against them, or by putting a finger in the auditory canal, we are struck more forcibly be the sound of the tuning fork than with our ears open. If one ear is closed and the other open, we hear a louder sound in the closed ear than in the open one. We observe the same thing if we close the right ear and apply the point of the tuning fork to the skin of the left temple; even if the tuning fork is nearer the left ear and the auditory canal and is much farther away from the right ear, it has a much stronger effect in that ear than in the left one, and vice versa.”

The tuning fork saw another use in 1855, when Heinrich Adolf Rinne (1819–1868) described another test in 1855 [104]: “A simple test shows us to what extent the normal conduction of sound by air and the tympanic membrane etc. prevails over conduction through the cranial bones, even for sounds which are produced by the oscillation of a solid body and are transmitted directly onto the skeleton. I apply to the upper incisors a tuning fork producing a sound and leave it in place until the sound which was at first very clear is no longer audible to me. I then move the tuning fork in front of the outer ear and hear the sound again very strongly. Only after a certain time does the sound stop here also. All the people with healthy ears on whom I have done this test have produced the same result […] If the patient hears the sound transmitted through the cranial bones for as long as or longer than the sound transmitted in the normal way, we conclude that part of the conduction system, including the membrane of the oval window, is diseased; this can also be caused by a motor affection of a nerve.” This test was introduced into practice by Johann Constantin August Lucae (1835–1911) from Berlin [105] and supported by Friedrich Bezold [106, 107] in the 1880s. Dagobert Schwabach (1846–1920) from Berlin completed these tuning fork tests with his own test [108].

In 1802, Christian Heinrich Wolke (1741–1825) developed the first “acuity meter” designed specifically to measure hearing [109]. Various other models and methods were then proposed, notably the use of a pocket watch [110]. The first audiometer was developed by the German otologist Arthur Hartmann (1849–1931) in 1878 [111]. His “Akumeter” combined the electrical tuning fork and the telephone receiver [112]. At the same time, others were produced in the United Kingdom and elsewhere. Since it was not commercially produced, it was not available and thus disappeared from history. In 1881, Arthur Hartmann wrote the textbook *Die Krankheiten des Ohres und deren Behandlung* (*The Diseases of the Ear and Their Treatment*; [113]), which had eight editions and translations into English, French, and Italian.

**Fig. 7:**
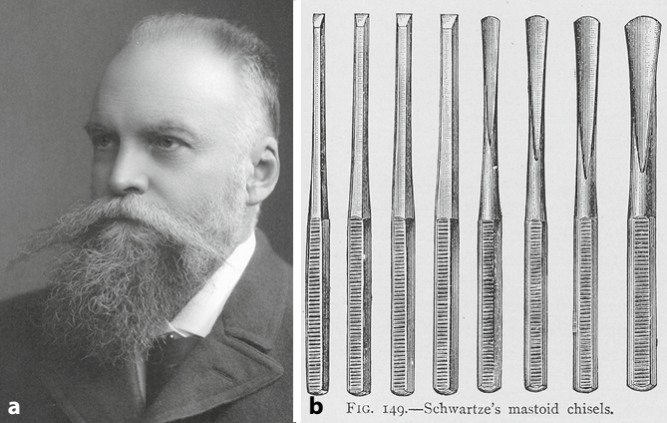
**a** Hermann Schwartze. (ORL Clinic Halle, Reprinted with permission © University Archive, Martin-Luther University Halle-Wittenberg, UAHW, Rep. 40, VI, Nr. 1 Bild 39, all rights reserved). **b **mastoid chisels. (From Dench’s textbook, 1896, [280])

In the second part of the nineteenth century, great progress was made in the field of ear therapy. In 1873 Hermann Schwartze [114] and his assistant Adolf Eysell (1846–1934) [115] of Halle proposed a new technique to open the mastoid by using a chisel and gouge, often called “modern mastoid operation” or Schwartze operation. Hermann Schwartze’s chisel was a short-bladed hand tool with a straight, beveled cutting edge and a plain handle that was struck with a mallet (Fig. 7). The gouge derived from the same instrument but had a concave, beveled cutting edge. Hermann Schwartze did not depict his instruments in his publications, but illustrations were published by colleagues. Hermann Schwartze described the advantages of the chisel and gouge over the trepan and other perforators. This new instrumentation enabled the creation of a larger, safer, funnel-shaped opening of the mastoid region down to the antrum for drainage of mastoid abscesses. It also expanded the indications for mastoid surgery, notably to include cases of chronic otitis.

Mastoidectomy rapidly became a routine operation [116]. Based on his extensive surgical practice requiring in-hospital treatment, he was able to move into the first hospital in Germany solely built for the specialty in 1884 with 25 beds for otology patients only (and a similar number for ophthalmology). Different types of mastoidectomy (cortical, radical, and modified radical) were developed mainly in Germany. During the winter of 1888–1889, two general surgeons from Berlin, Ernst Küster (1837–1930; [117]) and Ernst von Bergmann (1836–1907; [118]), read papers before the German Surgical Society in which the former recommended the removal of the posterior meatal wall and the latter the outer attic wall. Ernst Küster wrote: “The rational treatment must be based on the surgical principle that a diseased bony cavity should be opened up extensively, all diseased tissue removed, and the source of suppuration brought clearly to the light. The pus must nowhere be hindered in its outflow.” These principles were also further advanced notably in Germany by Ludwig Stacke (1859–1918) of Erfurt, who sought to combine the tympanum, attic, and antrum into one cavity, which facilitated inspection and dressing. Ludwig Stacke worked backward from the attic to the antrum [119], while some others worked forward from the antrum to the attic.

In 1889 Otto Körner (1858–1935) from Rostock suggested a modification of the radical operation that consisted in removing the upper posterior wall of the auditory canal and the outer wall of the antrum, leaving some of the outer attic wall in order to preserve the malleus and incus. Hermann Schwartze in 1893 also extended his own simple mastoidectomy to include removal of the posterior and superior meatal walls and middle ear contents. One of the problems with the earlier cases of radical mastoidectomy was that after healing, the cavity became inaccessible from the ear canal. To overcome this, meatoplasty was devised by Ludwig Stacke, Hermann Schwartze, and Rudolf Panse (1863–1942) of Dresden, among others. The enlarged meatus gave good access to the cavity and enabled closure of the postaural wound by primary suture. Before meatoplasty it was quite common for a deliberate postaural fistula to be created to facilitate dressing of the mastoid cavity.

There followed a period of approximately 7 years during which time many ears were automatically subjected to the radical operation and patients suffered from often unnecessary hearing loss. The same men who first advocated the radical procedure were themselves the first to attempt to save the ossicles and hence the hearing. They thus initiated the phase of conservatism aimed at the preservation of the middle ear function in those cases with only atticoantral disease and otherwise healthy middle ears.

The gradual realization that a fixed stapes alone could cause hearing loss encouraged certain otologists to propose freeing it in an attempt to improve hearing. This was done by Johannes Kessel (1839–1907) in Jena in 1876 [120]. He removed the columella from pigeons and the stapes from dogs, then stimulated their hearing, and observed that with the healing of the oval window, in which the stapes is normally situated, their hearing improved. He concluded that removal of the tympanic membrane and of the malleus and incus bones, combined with freeing of the stapes, could be a treatment for hearing loss. Two years later he examined a patient who had gone deaf after falling from a cart. The patient died and the autopsy showed a fracture across the horizontal semi-circular canal. Kessel concluded that the canal was part of the hearing function and that it had to be intact to allow hearing to be good. He deduced that, if the stapes became fixed, hearing could probably be improved by opening a semi-circular canal. He thus advocated fracturing the horizontal semi-circular canal and excising the fixed stapes. The oval window was then covered by a graft [121]. The effects on hearing were inconsistent. Johannes Kessel was also a precursor of radical mastoid surgery through an endaural approach [122, 123].

**Fig. 8:**
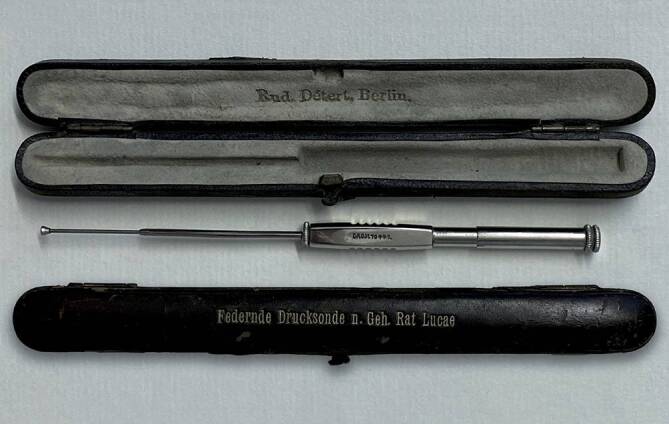
August Lucae spring probe. (Reprinted with permission © R. Mlynski, all rights reserved)

Also aiming to solve a fixation of the stapes, August Lucae in 1884 [124] introduced a new “method to mechanically treat chronic troubles of the mobility of the hearing organ transmission apparatus.” It consisted in the use of a springy pressure probe to directly mobilize the handle of the malleus (Fig. 8; [125]). Another idea was proposed by Karl Adolf Passow (1859–1926) [126] from Berlin, who, in 1897, reported to have made a window in the promontory and covered it with the tympanic membrane [127]. His patient noted a slight improvement in hearing. It precluded the fenestration operation.

The concept of a surgical repair of the tympanic membrane with a skin graft is usually credited to Emil Berthold (1836–1922) from Königsberg, in 1878 [128]: “The first step is to free the margin around the perforation and the lip-shaped epithelization of the margins of the perforation from the epithelium in order to change (render) these parts into a wound which enables the healing of a freshly excised piece of skin. For that purpose, I glue a court-plaster over the site of perforation so that the eardrum is covered by it still several millimeters distant from the perforation. After three days I remove this plaster. I harvest the piece of skin from the forearm. I introduce this piece of skin into the external ear canal and press it with its wounded surface over the margin of the perforation” [129, 130]. He termed his new technique “myringoplasty.”

In 1902, the term “cerebellopontine-angle tumor” was introduced by Richard Henneberg (1868–1962) of Berlin [131]. During these early years of the century, although the surgery of cerebral tumors was in considerable disarray, largely due to the publication of Ernst von Bergmann’s discouraging views on the subject, the first operations on suspected acoustic tumors were undertaken [132]. Fedor Krause (1857–1937) of Düsseldorf in 1903 reported an 83.8% operative mortality using the unilateral suboccipital approach [133]. Only 1 year later, Rudolf Panse in 1904 proposed that an approach through the labyrinth may allow for removal of an acoustic neuroma as large as a hen’s egg [134]. He defined the anatomical limits of this exposure as the lateral sinus, the jugular bulb, the carotid artery, and the temporal lobe. The facial nerve, he thought, should be sacrificed, but he did concede that with certain tumors it might be possible to re-route the nerve by mobilizing it from the geniculate ganglion to the stylomastoid foramen.

Otology was a fruitful specialty in Germany throughout the 19th century

During the first part of the nineteenth century, otology lectures were given—except for Gustav Lincke in Leipzig and Wilhelm Kramer in Berlin — essentially by surgeons and ophthalmologists such as Karl Gustav Himly (1772–1837) in the 1830s, and Ernst Ludwig Schillbach (1825–1898) in the 1850s, both from Jena [135], or Johann Adolf Winter (1816–1901) in Leipzig in the 1840’s. In 1852, Adolf Winter opened a private polyclinic for ear patients [13]. The first known academic lecturer titles (Privatdozenten, “PDs”) in otology were Edmund Traugott Adolf Dann (1805–1851) in 1832 [136] and Martell Frank (1810–1886) in 1849 [17], both in Munich. From 1859, many other otologists received their title as Privatdozent. In 1866, Salomon Moos (1831–1895) of Heidelberg was probably the first to become “extraordinary professor” in otology in Germany [137]. In 1878 [138, 139], in 14 of the 20 German universities, otology lectures were given by PDs or extraordinary professors. At the same time, 12 universities had a clinic or a subsidized polyclinic. In 1892, the German Otological Society was founded [140], while the first otological society was created in New York as early as in 1868 [141]. In 1902, all universities proposed lectures in otology [12]. In 1878, Anton von Tröltsch contacted the government to propose otology as a topic to be tested during the final examination for becoming a physician [138].

Throughout the 19th century, otology was a fruitful specialty in Germany. All its aspects were studied and developed, at the same time as numerous German innovations were implemented in the world of otology.

### Laryngology (pharyngo-laryngology and endoscopy)

The beginning of laryngology as a subspecialty is usually associated with the invention of the laryngeal mirror in 1855 [142], but most effectively it was in the 1880s, a time when it was possible to operate in the larynx with local anesthesia [143]. Laryngology was much less developed than otology, and some of the foundations of the subspecialty were established in the first part of the nineteenth century.

In anatomy, Ludwig Julius Caspar Mende (1779–1832) of Greifswald discussed in detail the motility of the vocal cords in 1816 [144]. The mechanism of the falsetto voice was a source of concern and investigation. Carl Lehfeldt (1811–1891) of Berlin in 1835 [145] offered an explanation that the falsetto tones arose from the larynx alone and that they were produced by vibration only of the edges of the vocal folds. The gross anatomy of the faucial tonsils had been studied and described from the earliest times. Albert von Kölliker, in 1852 [146], was the first to demonstrate the folds and depressions of the mucosa, the follicles in their walls and the epithelium. The finer structures of the lymphatic network, however, escaped the relatively feeble power of his microscope. Kölliker regarded them as part of the lymphatic system and related to the Malpighian corpuscles of the spleen. When Albert von Kölliker described the faucial tonsils, he mentioned the existence of similar tissue in the nasopharynx. Jacob Henle (1809–1885) of Heidelberg insisted that the “pharyngeal bursa” was a normal structure [147]. He already confirmed, in 1838 [148], the existence of different forms of epithelial covering. He identified pavement, cylindrical, and ciliated forms and found the latter types on the anterior wall of the larynx and on the posterior wall only immediately above the superior vocal ligaments. As early as in 1805, Johann Christian Rosenmüller (1771–1820; [149]) of Leipzig described the pharyngeal recess behind the ostium of the Eustachian tube, which bore his name [150]. In 1816, he also published a dissertation on the defect of the olfactory nerve [151].

In 1884, Heinrich Wilhelm Gottfried von Waldeyer-Hartz (1836–1921) of Berlin described the arrangement of lymphoid tissue around the junction of food and air passages [152] under the terms “tonsillar ring” or “lymphatic throat ring,” later eponymously known as “Waldeyer’s ring.” In 1868, Hubert von Luschka (1820–1875; [153]) of Würzburg provided a full description of the median and lateral recesses of the pharyngeal tonsil that belonged to Waldeyer’s ring. In the roof or vault of the pharynx, follicular glands are arranged [154], called “tonsil of Luschka” [155]. In 1885 Gustav Ludwig Tornwaldt (1843–1910) of Danzig described [156] the diverticulum of the pharyngeal tonsil, which was eponymously named “Tornwaldt’s pharyngeal bursa” [157]. It was first mentioned in some animals in 1840 [158].

The growing importance of laryngeal pathology was emphasized in 1829 by the publication of a book, *Die Pathologie und Therapie der Kehlkopfskrankheiten* (*Pathology and Therapy of Larynx Diseases*), which was devoted to laryngeal diseases [159], by Johann Friedrich Hermann Albers (1805–1867), an anatomist in Bonn. It contains separate sections on simple catarrh and chronic hypertrophic catarrh of the mucous membrane, of syphilitic and tubercular ulcerations, of disease of the cartilages, as well as growths and paralysis of the larynx. In 1836, a characteristic condition of the floor of the mouth, which usually arises from infection of the lower molar teeth and can give rise to respiratory obstruction, was described by Wilhelm Frederick Ludwig (1790–1865; [160]) and is still known as “Ludwig’s angina” (*angina Ludovici*). Wilhelm Frederick Ludwig died after developing an inflammation of the neck, raising the question that he might have died of “his own” condition [161].

Clinical examination of the upper aerodigestive tract found its “letters of nobility” in the second part of the nineteenth century. Philip Bozzini (1773–1809), working in Frankfurt/M. in 1806, described a double cannula with two mirrors placed at 45° at the end [162, 163]. The light was transmitted through one compartment and reflected from the mirror onto the parts to be examined. The image was received on the other mirror and reflected back to the eye through the second compartment. A wax candle provided the illumination, and the instrument that Philip Bozzini called the “Lichtleiter” was used to inspect a variety of canals including the larynx and the ear, although it is doubtful that he ever saw any part of the latter with this instrument. It launched a new era in the possibility to examine the upper aerodigestive tract, but it took more than a half century to be efficiently developed.

Credit for the first direct esophagoscopy is given to Adolf Kussmaul (1822–1902) of Freiburg, who in 1868, using a Desormeaux urethroscope elongated to 24 cm, managed to diagnose cancer of the thoracic esophagus [164–167]. He was unable to reach the cardia and so lengthened the instrument to 47 cm but despite using the services of a professional sword swallower there is no record that he actually entered the stomach. One of his pupils succeeded in safely introducing a rigid tube 13 mm in diameter into the esophagus of a healthy person. In the same year, Louis Waldenburg (1837–1881) of Berlin designed an esophagoscope, first consisting of a short gum elastic tube 8 cm in length [168, 169]. It was connected to a two-pronged fork, which enabled it to be secure while a laryngeal mirror was inserted into the mouth in order to see down the tube. He later designed a telescopic metal tube and attached a mirror to it.

The interior of the larynx was observed by direct examination for the first time in 1895

**Fig. 9:**
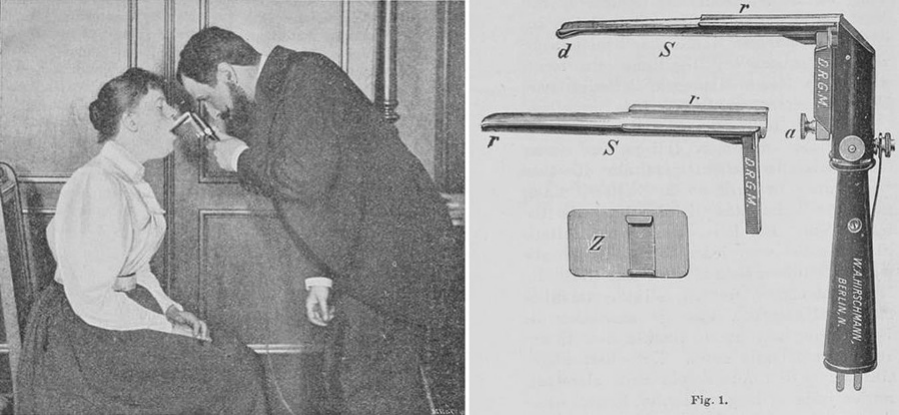
Kirstein (*left*) demonstrating the use of his autoscope (*right*) (From Kirstein’s 1895 publication, [171])

On April 23, 1895, Alfred Kirstein (1863–1922; [170]) of Berlin observed for the first time the interior of the larynx by direct examination [171, 172]. He used a flat spatula and Caspar’s electroscope, and with the patient’s head extended, he depressed the tongue and epiglottis, calling this examination “Autoscopie” (Fig. 9). After some experimentation, he concluded that indirect laryngoscopy was the best technique for viewing the anterior part of the larynx but that his direct method gave a better view of the posterior part of the larynx. He later abandoned the Caspar electroscope as it occluded the view and interfered with instrumentation. He substituted his own design of head lamp. Impressed by the work of Alfred Kirstein, Gustav Killian (1860–1921) of Freiburg [23], in 1896, began devoting his entire time to endoscopy. He adapted the esophagoscope, enabling him to perform a bronchoscopy in 1897 [173] and coined the term “bronchoscopy” [174]. Using tubes of an exterior diameter of between 9 and 10 mm, he was able to examine the main branches of the bronchi and their second and third divisions. Gustav Killian designed a short split laryngeal spatula to facilitate easier introduction of the tubes. Initially the procedure was performed with the patient seated on his specially designed chair. Later in 1911, he introduced the dorsal position for the patient and suspension laryngoscopy, which enabled him to free both his hands. Gustav Killian was the first to remove a foreign body (a bone) from the bronchus and in 1900 he succeeded, using a galvanocautery snare, in cutting in half a vulcanite dental plate that had become impacted deep in a patient’s esophagus. After the remarkable progress of endoscopy during the last 20 years of the nineteenth century, endoscopy made its marks in the first two decades of the twentieth century [175, 176]. Wilhelm Brünings (1876–1958) of Jena, who had been Gustav Killian’s chief assistant, introduced the idea of double extension tubes to bronchoscopes and esophagoscopes in 1908. His book *Die direkte Laryngoskopie, Bronchoskopie und Oesophagoskopie* (*Direct Laryngoscopy, Bronchoscopy, and Oesophagoscopy*) published in 1910 [177] was translated into English. Wilhelm Brünings was not only a designer of endoscopic instruments but also a pioneer in the teaching of this new specialty.

Gustav Killian coined the term “bronchoscopy” in 1897

The study of vocal cord movement was further aided by the first presentation in 1878 [178] of a stroboscope by Max Joseph Oertel (1835–1897) from Munich, which saw its final form in 1895 [179–181]. It allows one to analyze the vibration behavior of the vocal fold mucosa, making surgery of the vocal fold mucosa manageable.

**Fig. 10:**
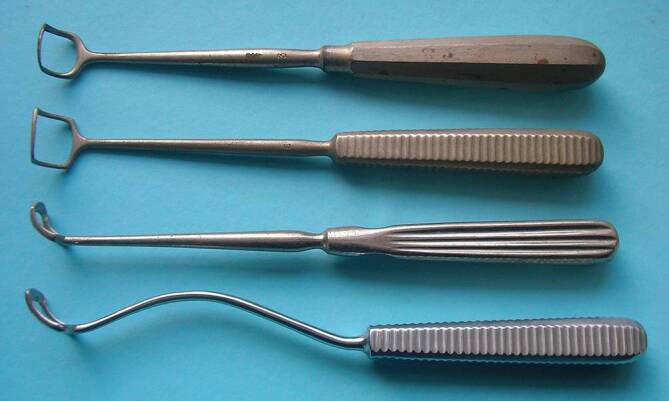
Beckmann’s curettes. (Personal collection, Dr. Lübbers. Reprinted with permission © W. Lübbers, Hannover, all rights reserved)

Therapy saw great progress in the second part of the nineteenth century. Tracheostomy was well established, as was tonsillectomy as a routine operation notably after the introduction of a kind of guillotine, the “tonsillotome” [182], by modifying an instrument to section the uvula in the 1820s. This instrument saw various modifications up until the beginning of the twentieth century. It was gradually replaced in the twentieth century by the “dissection technique,” with the patient placed on their back with a sandbag under the shoulders and with the head well extended. Nevertheless, due to an inadequate technique, tonsillectomy was regularly considered as “tonsillar massacre” [183]. Recognition of the importance of the adenoids, described in 1868 [184, 185], in the development of middle ear infections opened a new era in the comprehension of otitis. Removal of the adenoids quickly became a routine operation, first with a type of ring-knife and then with the curette. In 1886 Jacob Gottstein (1832–1895) of Breslau introduced the adenoid curette [186], which has since been modified by many surgeons, notably Hugo Beckmann (1861–1907) in Berlin (Fig. 10; [187]). The operation was initially performed without anesthetic but by the early 1890s general anesthesia was introduced.

Albrecht Theodore Middeldorpf (1824–1868) of Breslau described in *Die Galvanocaustik *(*The Galvanocaustic*) published in 1854 [188] the successful removal of a polyp, which was said to have arisen from the upper part of the larynx, using an incandescent platinum wire loop. He pulled the tongue forward with a sharp hook and guided the wire loop around the tumor with his fingers. In 1862 Victor von Bruns (1812–1883) in Tübingen claimed that [189] he had successfully removed a growth from his brother’s throat using forceps. In 1868, he published a series of 23 observations of laryngeal polyps [190]. Nevertheless, the extent of treatment that the laryngologist of the last quarter of the nineteenth century could offer was limited to the opening of abscesses, the removal of tonsils, and the endolaryngeal removal of polyps and other small tumors of the larynx. Caustic, astringent, or sedative solutions were applied to the larynx with a camel hair brush or syringed or sprayed into it; alternatively, astringent or sedative powders were blown in by an insufflator. Functional or hysterical loss of voice was treated by applying galvanic current to the vocal folds. The diagnosis between chronic laryngitis, syphilis, tuberculosis of the larynx, and malignant disease was always difficult and sometimes impossible, even for the experienced laryngologist. The introduction of cocaine as local anesthesia for the larynx led to a new therapeutic era.

The first laryngectomy was performed by Theodor Billroth in 1873

In 1854, Bernhard Rudolf Konrad von Langenbeck (1810–1887) of Berlin proposed to a patient the extirpation of the larynx for a malignant disease, but the patient declined [191]. Surgeons were now prepared to extirpate diseased organs such as the larynx, which was reported to be successfully performed for the first time by Theodor Billroth (1829–1894) from Bergen a. Rügen in 1873 in Vienna [192], creating a definitive opening of the trachea in the neck. The early laryngectomies were fraught with complications, the main one being aspiration. Some surgeons attempted to avoid complications by performing a preliminary tracheotomy several weeks before the excision of the larynx. In 1875, Bernard von Langenbeck realized that cervical lymph node metastases, if present, had to be removed with the primary tumor if the patient was to be given a chance of survival [193, 194].

In 1880, Max Schüller (1843–1907) in Greifswald published one of the first German books on tracheotomy, laryngotomy, and removal of the larynx [191]. One year later, Themistokles Gluck (1853–1942) of Berlin [195] suggested severing the trachea from the larynx and suturing it to the skin [196]. He favored removing the larynx from above and closed the pharyngeal defect before finally detaching the larynx from the trachea. This improvement was made in conjunction with his colleague Johannes Soerensen (1862–1939; [197]). Bernhard von Langenbeck, Themistokles Gluck, and Johannes Soerensen regularly examined the vessels of the neck and removed lymph node metastases together with the sternomastoid muscle, the internal jugular vein and, on occasion, the carotid artery. Neck dissection was thus included in surgical treatment.

In the second part of the nineteenth century, vocal rehabilitation methods included the development of esophageal speech and the use of mechanical vibrators. The first successful reports of pharyngeal speech in Germany came from Hans Schmid (1853–1896) of Stettin, in 1888 [198, 199], and Jacob Gottstein in 1900 [200]. Only Julius Wolff (1836–1902) of Berlin in 1893 was able to create different sounds in his artificial larynx as a result of a rubber tongue that could be elongated or shortened with the turn of a screw [201]. After the remarkable development of esophageal and bronchial endoscopy during the last 20 years of the nineteenth century, endoscopy made its mark in the first two decades of the twentieth century, also becoming therapeutic. It was included in the domain of most OHNS specialists.

In the 1860s, the first lectures in laryngology were essentially concentrated on the correct use of the mirror to examine the larynx. These lectures were usually called “laryngoscopy.” At the same time, the first academic lecturer PDs in laryngology were received, e.g., by Georg Richard Lewin (1820–1894) in Berlin in 1862, becoming extraordinary professor 6 years later [16]. He was followed by many others in the different German universities. The lectures progressively changed to become true laryngology lectures in the 1880s, often associated with lectures on rhinology. In 1887, one of the first independent polyclinics for laryngology and rhinology opened in Berlin. The Berlin Laryngological Society was founded 2 years later under Bernhard Fraenkel (1836–1911), followed by the German Laryngological Society in 1894, while the first laryngological society was created in New York as early as in 1873 [202]. In 1908, only six German universities had a laryngology clinic [203].

Contrary to otology, laryngology saw its main development in Germany in the second part of the nineteenth century. Different German protagonists played an important international role, notably in the area of examination and treatment.

### Rhinology

Rhinology is often considered as the poor relative in the history of OHNS. It was usually associated with laryngology. Nevertheless, it tried to find its own way at the end of the nineteenth century.

In anatomy, an early reference to the erectile tissue [204] in the nose was made by Otto Ludwig Bernard Kohlrausch (1811–1854) of Hannover, who in 1853 [205] spoke of cavernous tissue of the posterior border of the inferior turbinate.

There was important progress in clinical examination with the possibility to access the posterior part of the nose. Friedrich Eduard Rudolf Voltolini (1819–1889) of Breslau [206] introduced posterior rhinoscopy in 1859 and invented an oxyhydrogen incandescent light to aid examination of the ear and larynx. He actively pursued posterior rhinoscopy as an aid to the passage of the eustachian catheter. In 1861, he published the first edition of his textbook *Die Rhinoskopie und Pharyngoskopie *(*Rhinoscopy and Pharyngoscopy*; [207, 208]).

**Fig. 11:**
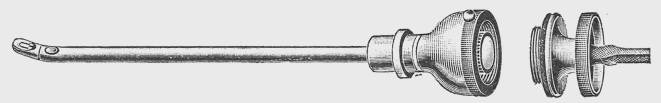
Hirschmann nasal endoscope, 1905. (From Reiniger-Gebbert & Schall. Elektro-Med. Apparate, [281])

It was only 6 years later, when radiology was discovered, that Max Scheier (1864–1921) of Berlin was quick to see its value in the diagnosis of sinus disease. His pictures of 1896 were neither perfect nor convincing, but they showed that—given further development—the technique was promising. He published them in 1897 [209, 210]. The name of W. Alfred Hirschmann of Berlin is associated with the introduction of sinoscopy since 1901 [211, 212] in Germany. He managed to reduce a Nitze’s cystoscope to a diameter of 4.0 mm in order to study the middle meatus and sinus ostia (Fig. 11). He originally entered the antrum through a molar tooth socket but he also trephined the canine fossa. Diagnostic puncture of the maxillary antrum attracted some authors in close succession. Hermann Krause (1848–1921) of Berlin in 1887 [213] modified Mikulicz’s trocar by adding a cannula to permit antral lavage.

In the mid-nineteenth century there was a great difference of opinion concerning the pathology of nasal polyps. Maximilian Joseph von Chelius (1794–1876) in Munich still supported the concept of the Middle Ages, that polyps result from a local infiltration of mucous membrane with serum [214]. In 1854 some considered that nasal polyps were adenomatous swellings and Rudolf Virchow in 1863 called them “myxomata.” It was then suggested that nasal polyps arose from a chronic infection of the ethmoid air cells.

The plexus of veins situated on the anterior part of the cartilaginous septum was identified as a source of epistaxis by Carl Michel (1843–1930) of Cologne in 1874 [215], and Wilhelm Kiesselbach (1839–1902) of Erlangen in 1880 [216] and 1884 [217]. This area was later named “Kiesselbach’s plexus.” To treat epistaxis, the German Johann Peter Frank (1745–1821) appears to have been the inventor, as early as in 1807, for he was the first to devise a special instrument (balloon tamponade if it can be called so) to bring pressure to bear directly on the walls of the nasal fossae. He introduced into the nose a piece of dried hog’s intestine, tied at the distal end, and then injected water into the open-end projecting from the nostril, tying up the gut as he withdrew the syringe [218, 219]. Over time, the pig intestine became a rubber fingerling or a condom, which had just come on the market at the end of the nineteenth century.

In 1886 Karl Konstantin Heinrich Ziem (1850–1917) of Danzig stressed [220] that the origin of sinus disease was to be found in the nose. The term “sinusitis” appeared in the rhinology literature at the turn of the 1890s. It became rapidly used worldwide to describe an inflammation of the paranasal sinus. In 1882, Wilhelm Hack (1851–1887) of Freiburg im Breisgau raised the awareness of laryngologists to the association of upper airway disease with asthma [221].

Surgery made big strides with the refinement of rhinoplasty and septoplasty

Surgery took a big step forward with the development and refinement of rhinoplasty and septoplasty. Carl Ferdinand von Graefe (1787–1840) of Berlin introduced the term “rhinoplasty” in his book published in 1818 [222] and in reviving the Italian method by combining its best features with the Indian method, thus creating a European technique and becoming the founder of modern plastic surgery. He was succeeded in Berlin by Johann Friedrich Dieffenbach (1792–1847), who emphasized constructive rather than ablative nasal surgery: “The nose is man’s most paradoxical organ. It has its root above, its back in front, its wings below and one likes best of all to poke it into places where it does not belong.” Friedrich Dieffenbach remarked that “only very young and very healthy people with great courage can endure it and the surgeon must have great experience” [223, 224]. He also proposed using gold removable supports to keep the nostrils open. In 1822, Heinrich Christian Bünger (1782–1824) of Marburg used the Indian technique with a flap from the leg [225]. Friedrich Dieffenbach was one of the first to use ether anesthesia. He introduced the “endonasal” approach to rhinoplasty and his *Die Operative Chirurgie* (*Operative Surgery*) published in 1845 [226] contains over 100 pages on flap reconstruction of noses, including V‑Y advancements and Z‑plasties of the nasal base. He also tried to include a gold plate to sustain a saddle nose. By the end of the nineteenth century, the principles and practice of rhinoplasty were well established [227]. In 1898 Jacques Lewin Joseph (1865–1934) of Berlin introduced modern rhinoplasty [228] with the publication of his paper, “Ueber die operative Verkleinerung einer Nase” (“Operative reduction of the size of the nose”; [229]). He was an orthopedic surgeon, and the patient described was a male suffering from depression, on whom Jacques Joseph performed a large reduction rhinoplasty. It was done through an external dorsal V‑shaped incision, with excision of redundant dorsal skin. Jacques Joseph was a gifted illustrator who analyzed and classified rhinoplasty surgery and invented surgical instruments. In 1904, he began performing rhinoplasty through intranasal rather than external incisions, thus establishing a series of techniques that have been used by generations of rhinoplasty surgeons. His book *Nasenplastik und sonstige Gesichtsplastik* (*Rhinoplasty and Facial Plastic Surgery*), published in two parts between 1928 and 1931 [230] and later translated into English, became a classic text. He developed numerous instruments to perform his surgery [231]. The refinements and introduction of facial plastic surgery were facilitated by the great contribution of Jacques Joseph. The term “plastic surgery” was introduced in 1838 by Eduard Zeis (1807–1868) in Marburg in his *Handbuch der Plastischen Chirurgie* (*Textbook of Plastic Surgery*; [232]). In 1863, Eduard Zeis published the first exhaustive repertory of references in the history of rhinoplasty [233].

Ludwig Grünwald described an intranasal approach to the ethmoid sinuses

In 1834 Conrad Johann Martin von Langenbeck (1775–1851) of Göttingen (the uncle of Bernhard von Langenbeck) described a method of shaving down acute spurs and angulations of the nasal septum [234] and exostoses on the septal wall [235]. Others recommended complete removal of the deviation using punch forceps. Most of these procedures inevitably exchanged a septal deflection for a septal perforation. The idea of removing the deflected cartilage and bone submucosally came simultaneously to a number of independent workers in the mid-nineteenth century. Wenzel von Linhart (1821–1877) of Würzburg protected the nasal mucosa of both sides in practicing the resection of the septum in 1862 [236]. Since 1879, Arthur Hartmann used a raspatorium to separate the nasal mucosa from the septum [237]. The year 1882, however, definitively marked the beginning of the codification of submucous resection of the septal wall with specially designed instruments [238]. Very similar procedures were recommended by Robert Krieg (1848–1933) of Stuttgart in 1886 [239, 240] and Georg Boenninghaus in 1895 [241]. The latter was the first to publish long-term results. Gustav Killian’s submucous resection operation [242] gained popularity throughout the world, but it became apparent that problems could occur with the anterior part of the septum, mostly in the form of supratip depression and columellar retraction. The concept of a “septoplasty” operation gradually developed, where the cartilaginous septum, still attached to its mucoperichondrium, was mobilized and repositioned in the midline [243–245].

The significance and surgical drainage of the maxillary antrum were fully explored in the seventeenth and eighteenth centuries, but were ignored and neglected most of the time. On the whole, little new knowledge was added until the beginning of the development of modern rhinology in the early 1880s. Max Schaeffer (1846–1900) from Bremen performed endonasal ethmoid surgery and transnasal sphenoid surgery in 1885, easily piercing the sphenoid anterior wall with a sharp spoon probe [246]. The textbooks of the day contained few pages on sinus disease. There were, however, a number of notable exceptions. Ludwig Grünwald (1863–1927) of Munich published *Die Lehre von den Naseneiterungen *(*Textbook on Nasal Suppuration*) in 1893 [247]. It established the subject of sinus disease on a secure foundation. He described an intranasal approach to the ethmoid sinuses, having first prepared the nose with cocaine. His design of forceps is still in use. Ludwig Grünwald classified ethmoid infection into a “closed type,” accessible via the intranasal route, or an “open type,” where the infection had spread to involve the orbit or frontal sinus, which required an external operation. Bernhard Moritz Karl Ludwig Riedel (1846–1916) from Jena indicated, in a supervised thesis by his pupil Heinrich Schenke (1868–1935) in 1898 [248], a radical frontal sinus operation from the outside with removal of the facial and orbital sinus wall, which led to considerable disfigurement of the face. It was indicated only if the sinus was very small and narrow, and later named “Riedel’s operation” or “Riedel procedure” [249].

Various modifications of sinus surgery were made, mainly related to the formation of an intranasal opening and transplantation of mucosal flap into the antrum, as suggested by Georg Boenninghaus (1860–1945) of Breslau and Alfred Denker (1863–1941) of Munich [250]. Hermann Kuhnt (1850–1925) of Jena, Königsberg, and Bonn, aimed to remove the whole of the anterior wall of the sinus and obliterated it by allowing the skin to sink into it [251]. This produced an unsightly deformity. An even greater deformity resulted from the procedure suggested by Georg Limburg in Jena, in which the inferior and anterior walls were removed [252]. In an effort to avoid this, Gustav Killian in 1900 described an incision through the eyebrow and preservation of the supraorbital bridge [253]. He achieved a complete exposure of the frontal sinus and by prolonging the incision inferiorly he was also able to remove the ethmoid air cells, thus overcoming one of the shortcomings of the Ogston–Luc procedure. Albert Jansen (1859–1933) of Berlin in 1902 [254] proposed removal of the frontal sinus floor and preservation of the anterior wall with exenteration of the adjoining ethmosphenoid system.

**Fig. 12:**
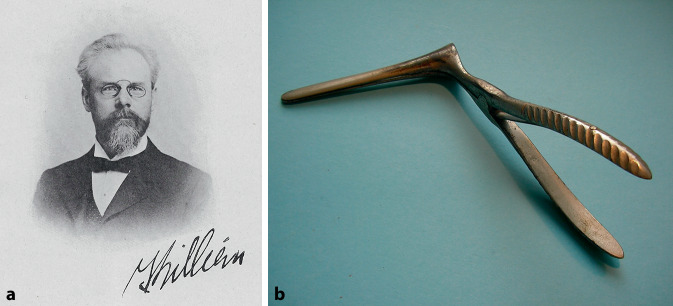
**a** Gustav Killian. (From Moure 1908, [282]). **b** Gustav Killian’s nasal speculum. (Personal collection, Dr. Lübbers. Reprinted with permission © W. Lübbers, Hannover, all rights reserved)

The osteoplastic flap operation [255] was originally described by Karl Wilhelm Schönborn (1840–1906) of Würzburg in 1894 [256], Oskar Brieger (1864–1914) of Breslau in 1895 [257] and Ernst Winckler (1860–1916) [258] of Bremen in 1904 [259]. In 1911, Gustav Spiess (1862–1948) of Frankfurt described a transeptal approach to the pituitary gland [260]. Gustav Killian was also very active in nasal surgery (Fig. 12). In 1876, Bernard Fränkel (1836–1911) from Berlin wrote one of the first German chapters dealing exclusively with rhinology [261]. In 1888, Rudolf Voltolini published *Die Krankheiten der Nase und des Nasenrachenraumes *(*The Diseases of the Nose and Rhinopharynx*; [262]), which became a very popular textbook in Germany. A few years later Carl Zarniko (1863–1933) in Hamburg published another textbook *Die Krankheiten der Nase *(*The Diseases of the Nose*; [263]).

Compared with otology and laryngology, rhinology was considered to be the “poor relative” of otorhinolaryngology in the nineteenth century. No specific rhinology clinics were recognized, and nearly no academic lecturer positions in rhinology were distributed. Nevertheless, rhinology saw an important development in Germany in the second part of the nineteenth century, notably, its surgical part.

## Creation of ORL specialty in the 20th century

### Foundation of ORL structures

The creation of the specialty of ORL was the amalgamation of otology, laryngology, and rhinology. It was marked by the foundation of the first ORL hospital departments and university lectures and chairs, the organization of the first specific national and international conferences, and the publication of the first ORL journals and books. It is outside the scope of this manuscript to go into too many details, but one aspect will be discussed here, i.e., the foundation of the first academic structures. Concerning books, it is worth mentioning that the German ORL textbooks appeared at the turn of the twentieth century. One of the first, in 1901, was *Anleitung zur Diagnose und Therapie der Kehlkopf‑, Nasen-, und Ohrenkrankheiten* (*Guide to the Diagnosis and Therapy of Diseases of the Larynx, Nose, and Ear*), by Richard Kayser (1854–?) from Breslau [264], the 16th edition being published in 1928. Interestingly, in 1909 Otto Körner published a second-edition textbook,* Lehrbuch der Ohren, Nasen- und Kehlkopf-Krankheiten *(*Textbook of Diseases of the Ear, Nose and Throat*; [265]). The first edition deals only with otology [266]. It is a good example of this amalgamation. The first German ORL-specific instrument catalogues were found at the same time, notably the Windler in 1893 [267] and Détert in 1901 [268], both in Berlin. Many instruments were eponymously named notably after Arthur Hartmann, Anton von Tröltsch, August Lucae, Bernhard Fränkel, Rudolf Voltolini, Hermann Krause, Berhard von Langenbeck, and Hugo Beckmann, to mention a few. New companies, such as Fischer in Freiburg im Breisgau and Pflau, again in Berlin, completed the list.

### Academic associations

**Fig. 13:**
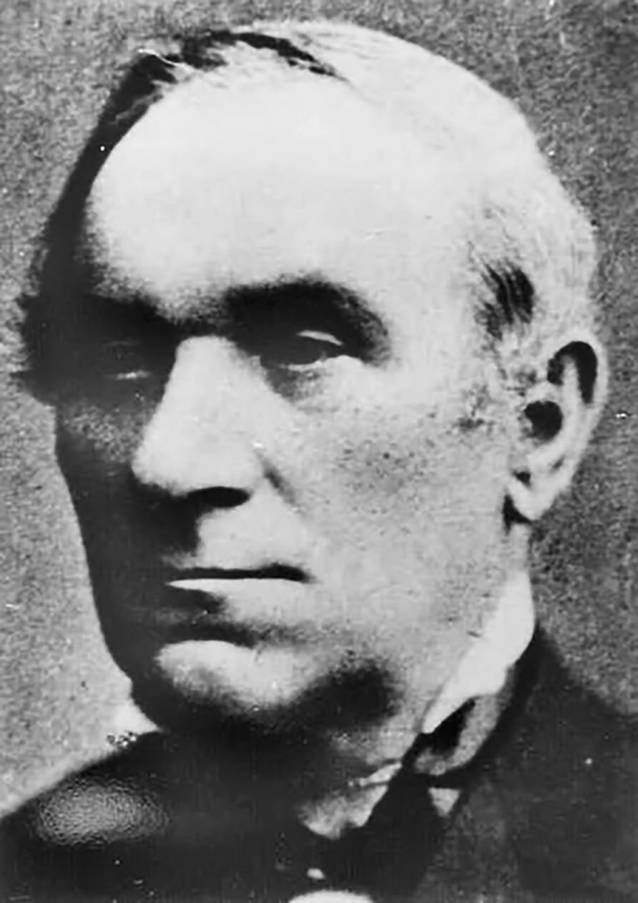
Rudolf Voltolini. (From [283])

**Fig. 14:**
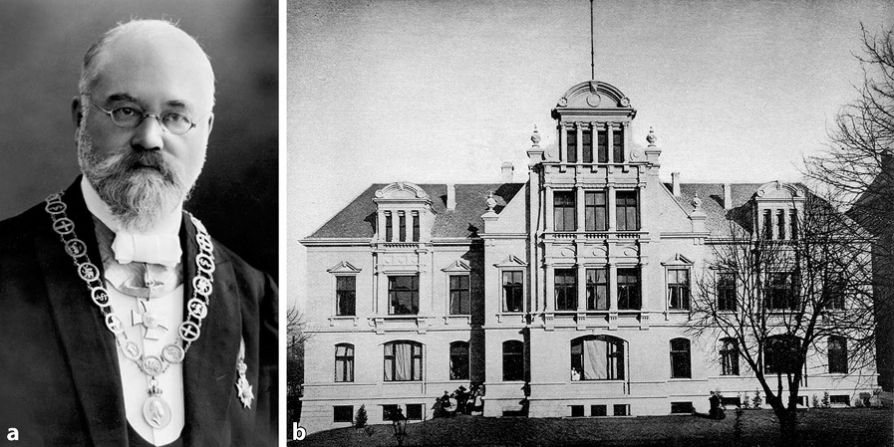
**a** Otto Körner as Rector magnificus 1913/14 of the University of Rostock. (Reprinted with permission © University of Rostock, all rights reserved). **b** the first ORL clinic in Rostock 1899. (From [284], reprinted with permission © ORL clinic, University of Rostock, all rights reserved)

As in many countries, there was not a linear route leading to the creation of the ORL specialty. It began with some solitary spots, which progressively came into contact through meetings and local societies. In 1860, Rudolf Voltolini in Breslau (Fig. 13) obtained his PD in otology and laryngoscopy, the first of its kind in Germany. Eight years later, he was promoted to extraordinary professor for otology and laryngoscopy by the Breslau Medical Faculty [269, 270]. Interestingly, his main publications dealt with rhinology. At the same time, otology started becoming a solid specialty with the promotions of many PDs in the 1860s. Laryngology was academically not so advanced, with the same thing happening only two decades later. Thus, the official academic association of otology with laryngology had to wait more than two decades to be seen again: This occurred at Rostock University, in 1883, with Johann Christian Lemcke (1850–1894). In 1884, he moved his activities to private practice. He received his PD in otology and laryngology in 1885 and from 1889 he had a room in the university polyclinic. In recognition of his achievements, Lemcke’s practice became “University Polyclinic for Ear, Nose and Throat Diseases” in 1891 [271]. Only as associate professor in 1893 did he receive financial support, but he died 1 year later. His successor Otto Körner went a step further, becoming extraordinary professor in ORL in 1894, which, from the beginning, was salaried by the university. In 1899, he had the opportunity to open a university clinic for ORL in a new construction [272], the first of its kind in Germany (Fig. 14). In 1901, he received the first German title of ordinary professor of ORL [273].

### Uniting the fields of otology, laryngology, and rhinology

But not everywhere was as simple as in Rostock. In many universities, heated discussions for and against a unification of otology, laryngology, and rhinology took place. Within the professional group of doctors who had set up a private practice, a fusion of the fields in question had already been taking place informally with some frequency. However, these fields lacked influence and did not carry any weight within university faculties. Many called for their acceptance in medical programs of study and examination regulations, but each individual discipline and its representatives did not possess enough influence within faculties to be able to assert their interests [30].

The first attempts to unite otology and rhino-laryngology were made in 1878

The first attempts to unite otology and rhino-laryngology were made in 1878 in Kassel during the 51th meeting of the German natural scientists and physicians. The new 20th section of laryngology and rhinology decided to unite with the 19th section, otology. This union took place, joint meetings were held, and rhinology was dealt with in joint sessions. At the 58th meeting in Strasbourg in 1885, the sections had again been separated.

**Fig. 15:**
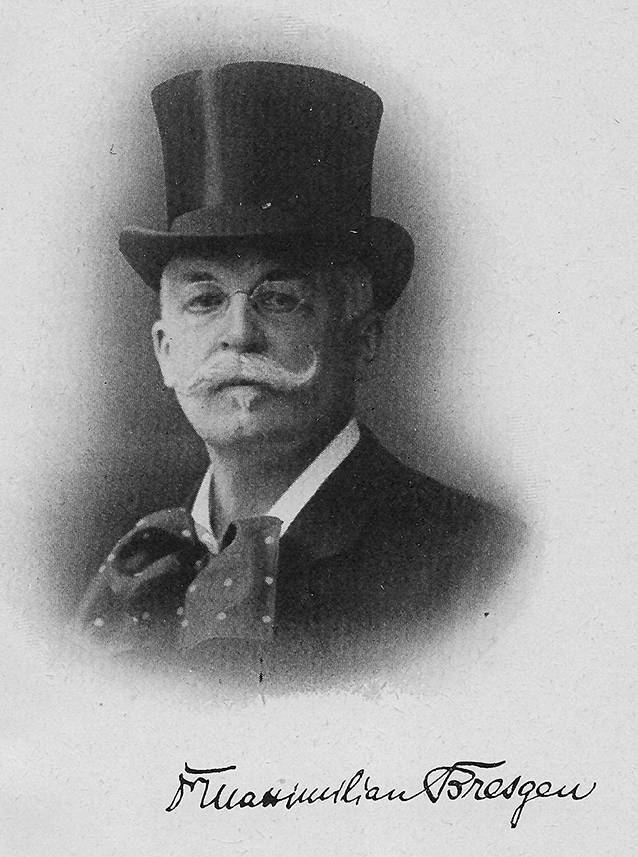
Maximilian Bresgen. (From Moure 1908 [282])

**Fig. 16:**
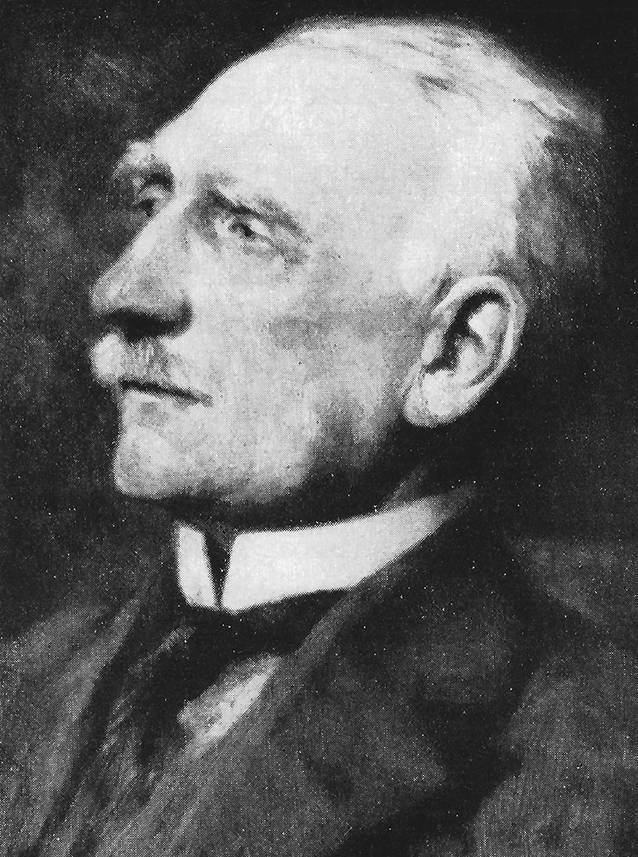
Adolf Passow. (From Kindler, 1956 [16])

Maximilian Bresgen (1850–1915) in Frankfurt (Fig. 15) and Adolf Passow (Fig. 16) were fervent supporters of the amalgamation. In 1883, Maximilian Bresgen asked for training of medical students in the special subjects with the creation of corresponding chairs [11, 274], foreseeing, that there would be a union of laryngo-rhinology with otology both in education, at congresses, in practice, and at universities. From his point of view, otology would subordinate to rhino-pharyngology. Given the abundance of patients and pathologies, it may be necessary, in large universities, to separate rhino-laryngology and otology. On the contrary, in small universities, one professorship should represent the three subjects! In 1908 Adolf Passow gave a lecture to the German Otological Society titled “Otology and laryngology, union or separation?” [203, 275]. “Laryngology and otology are two domains which have outgrown their differing origins and developed into important subdisciplines of medical science. In the beginning, they were separate, but it was an inevitable side-effect of their further development that they came closer to one another […] Rhinology and pharyngology served as their connecting links. There is not one otologist or laryngologist who can accomplish much of note without being as well versed in both of these disciplines as he is in his own field of specialization.” He also mentioned the power struggle within faculties: “If one calmly considers this matter, one must concede that faculties are correct in their opinion that ophthalmology, otology, laryngology and dermatology should not possess as much significance as the main disciplines.” In his opinion, an amalgamation of the study of medicine was a worthy aim and further fragmentation posed a threat to the development of such study.

Hermann Schwartze and Gustav Killian were notable opponents. Hermann Schwartze presented his opposition in discussing a lecture by Johann Ambrosius Barth (1852–1936) held in Leipzig in 1899 for his new position as director of the new clinic and polyclinic for ORL [276]: “I am convinced that otology, as well as ophthalmology, must be taught and worked as an independent discipline at the universities, if it is to prosper. Already now it does not grow up to such an extent that it needs the undivided power and time of an individual to represent it exhaustively. But on the other hand, it offers to be represented exhaustively; however, it offers such problems and tasks to be solved that the connection with a discipline like laryngology, which has no contact at all with otology, can only be a hindrance. The situation is different with rhinology, because of the multiple etiological relations of the diseases of the nose and the etiological relationships of the diseases of the nose and the ear cannot be neglected by otologists.” At a meeting 2 years later, Hermann Schwartze protested against this association [277]; only August Lucae supported him [278]. Gustav Killian’s arguments were predominantly based on the deterioration of treatment that he believed would assuredly follow from any process of amalgamation. He argued that the academic instructor could not be competent in either of the two specialized disciplines to the extent that was necessary for the advancement of science and teaching [30].

On November 9, 1918, both boards of the German Otological Society and the Association of German Laryngologists decided to establish the Society of German Otolaryngologists. However, this was not possible, due to the revolution just taking place in Germany. Thus, both boards met again during the meeting of natural scientists and physicians in Bad Nauheim and set May 12, 1921 as the date of foundation. The founding meeting was chaired by Georg Boenninghaus as the representative of the laryngologists. In his speech, he expressed the injustice deeply felt by many Germans with the war guilt allocations and the consequences of the Treaty of Versailles. The recently deceased Gustav Killian was particularly commemorated. He had helped to propose the unification to the laryngologists. Originally, he was a fierce opponent of a merger; however, it then applied only to his laryngology clinic in Berlin, where his otology colleague, whom he did not love, had always been pushing with force for the unification into one overall specialty, of course also at his clinic in Berlin. This behavior clearly shows the difficulties and contradictions that still existed at the time of the foundation of the Society of German Otorhinolaryngologists. This new national society was founded much later than most of the other European national societies, for instance, in France in 1882, Spain in 1886, Italy in 1892, The Netherlands in 1893, or Switzerland in 1912 [31].

## Conclusion

The scientific and clinical developments during the period described in this article were crucial and consequently allowed for the foundation of the German Society of Otorhinolaryngology, Head and Neck Surgery on solid ground. Germany played an important role in the development and progress of otorhinolaryngology internationally in the nineteenth century with such great contributors as Anton von Tröltsch, Hermann Schwartze, Otto Körner, Rudolf Voltolini, and Gustav Killian to mention a few.

## Footnotes

1 Tröltsch A. Die Untersuchung des Gehörorgans und Trommelfells. Ihre Bedeutung. Kritik der bisherigen Untersuchungsmethoden und Angabe einer Neuen. *Dtsch Klin* 1860;12:113–115, 1860;13:121–123, 1860;14:131–135, 1860;15:143–146 and 1860;16:151–154.

2 Chladni EF. Entdeckungen über die Theorie des Klanges. Leipzig: Weidmann, 1787 [101]. Chladni EF. *Die Akustik*. Leipzig: Breitkopf & Härtel, 1802 [102].

## Declarations

For this article no studies with human participants or animals were performed by any of the authors. All studies performed were in accordance with the ethical standards indicated in each case.
